# The use of accelerometers to improve clinical outcomes in patients with implantable cardioverter defibrillators or at high risk of sudden cardiac death

**DOI:** 10.12968/bjca.2024.0068

**Published:** 2025-01-02

**Authors:** H Cheng, J Hill

**Affiliations:** University of Lancashire

## Abstract

The Implantable Cardioverter-Defibrillator (ICD) is pivotal in preventing sudden cardiac death, monitoring cardiac activity, and restoring normal sinus rhythm with electrical shocks. With sudden cardiac death accounting for nearly half of all cardiac-related fatalities, ICDs significantly reduce mortality in high-risk patients. When integrated with accelerometers, ICDs also track physical activity (PA), which helps predict cardiac support needs and the likelihood of hospitalisation. Increased PA following ICD implantation has been associated with improved clinical outcomes. Wearable accelerometers show promise for assessing individual physical behaviour (PB), potentially serving as prognostic predictors of future cardiac events. This commentary critically evaluates the recent systematic review by Kolk et al. (2022), which examines the association between PB and key clinical outcomes in patients with ICD or at risk of sudden cardiac death.

## Introduction

An implantable cardioverter-defibrillator (ICD) is a battery-powered device implanted under the skin for continuous cardiac monitoring, detecting any life-threatening arrhythmias, and prompt restoration of normal sinus rhythm via electrical shocks when necessary (Mirowski 1985). Sudden cardiac death accounts for approximately half of all cardiac-related deaths (Wong et al. 2019), therefore, ICDs serve as a significant preventative measure, reducing cardiac mortality among survivors of sudden cardiac death and patients at high risk of ventricular arrhythmias (Friedman et al. 2022).

Modern ICDs are designed to include a device-embedded accelerometer, which facilitates the detection of changes in physical activity (PA) (Kolk et al. 2022). It has been suggested that increased PA, often achieved through cardiac rehabilitation, is associated with a reduced risk of further cardiac complications and improved outcomes in patients with heart failure (Atwater et al. 2021)). Conversely, low PA following ICD implantation has been linked to an increased risk of mortality, ICD shock, and hospitalisation (Zhao et al. 2017).

Therefore, accelerometer-based methods have the potential to provide detailed insights into individual physical behaviour (PB), serving as a more precise indicator of clinical deterioration in cardiac health (Kolk et al. 2022). PB encompasses various dimensions, including PA, sleep patterns, daily activities, postures and sedentary behaviours (Bussmann and van den Berg-Emons 2013). A recent systematic review by Kolk et al. 2022 assessed the association between PB and clinical deterioration, leading to ICD therapy, heart failure hospitalization, and mortality (Kolk et al. 2022).

### Aim of commentary

This commentary aims to critically appraise the methods used within the systematic review by Kolk, et al. (2022) and expand upon the findings in the context of clinical practice.

### Critical appraisal and methods of the review by Kolk, et al. (2022)

Using the Joanna Briggs Institute (2017) critical appraisal tool for systematic reviews, 3 out of 9 criteria were deemed satisfactory for this review (Aromataris et al. 2015). See Table 1 for the full critical appraisal.

Firstly, from a methodological standpoint, the review posed a non-specific research question. When designing a systematic review, it is helpful to structure the question around one of the standardised systematic review types (Munn et al. 2018). Based on the review question and the results, it appears that the review aimed to assess both prognostic and diagnostic test accuracy simultaneously (Campbell et al. 2015; O’KeeffeGreene and Kearney 2014). This lack of clarity complicates the appraisal of certain methodological processes, as most methods are critically appraised in context to the specific aims of the review. Due to this mixture of methodological designs, it raises concerns about the appropriateness of using the Quality in Prognosis Studies (QUIPS) tool to assess the risk of bias, as this tool is not designed to assess test accuracy studies (Hayden et al. 2013). Consequently, the comparison of accuracy outcomes between device-embedded accelerometers and validated wearable accelerometers has not been adequately assessed in the context of potential bias.

The second area of concern was related to the search strategy and the limited number of databases searched. Within the search strategy, there was no clear rationale for why the review only searched from 2000 onwards. While this may be justifiable if certain technologies were introduced after this time, this was not stipulated within the paper. Additionally, the limited search raises concerns about potential indexing bias in the resources, which could result in important papers being missed. Previous recommendations for healthcare-related subjects suggest that at a minimum, essential databases such as Embase, MEDLINE, Web of Science, and Google Scholar should be included for comprehensive searches (Bramer et al. 2017).

The third area of concern was regarding data extraction. While the review indicated that two reviewers were involved in the data extraction process, there was no clear statement confirming that this was done independently. The current gold standard for data extraction is that it be conducted independently by two reviewers (Higgins et al. 2021). Thus, without independent verification, there is a risk of potential errors in the data extraction process.

The final area of concern pertained to the methods of synthesis and the assessment of publication bias. Concerning the synthesis methods, effect estimates for directional association were not always presented as ratios. This omission made it challenging to ascertain whether the directional association was representative of all the studies assessed or only those with a specific direction of association. Moreover, while there appeared to be consistency in the reporting of hazard ratios across various outcomes, no attempts at meta-analysis were undertaken. No explanation was provided for why this procedure was not conducted, nor were any breaches of meta-analysis assumptions addressed.

## Results of the review by Kolk, et al. (2022)

After removing duplicates, 4222 studies were identified, of which after screening, 52 studies were included in the systematic review. Of these studies, 22 studies were identified as having a low risk of bias, 19 as having a moderate risk, and 8 as having a high risk of bias.

Among the studies which measured device-embedded accelerometers, there was an association between low physical activity following device implantation and increased risk of mortality (5 studies, n = 101,617, 12–31 months), hospitalization (3 studies, n = 1,715, 15–36 months), combined hospitalization or mortality (3 studies, n = 1,715, 15–36 months) atrial arrhythmias (1 study, n = 770, 25 months) and ICD shock (1 study, n = 4,057, 1 month). A similar association was observed between low physical activity, measured using a wearable accelerometer, and an increased risk of hospitalization and mortality (3 studies, n = 286, 12–36 months).

Additionally, declining physical activity was associated with increased risk of mortality (4 studies, n = 126,234, 26–28 months), hospitalization (5 studies, n = 3,522, 11.7–17 months), combined hospitalization or mortality (3 studies, n = 1,715, 15–36 months), atrial arrhythmias (1 study, n = 770, 25 months), and ICD shock (1 study, n = 4,057, 1 month).

In the studies that utilized device-embedded accelerometers, several factors were identified as contributing to reduced physical activity. These included seasonal variation (1 study, n = 102, 12 months) and pandemic lockdown (1 study, n = 24, 80 days), both of which were associated with decreased physical activity. Conversely, implantable cardioverter-defibrillator placement (2 studies, n = 2,944, 12–22 months) and the onset of atrial fibrillation (1 study, n = 266, 51.6 months) were associated with a decreased risk of physical activity reduction.

For studies using wearable accelerometers, several factors were identified as associated with physical activity. Heart failure patients exhibited lower mean activity levels compared to healthy adults (1 study, n = not reported in the systematic review [N/R], N/R months). Unfortunately, while other outcomes related to wearable devices were reported, there were significant inconsistencies and a lack of data concerning important variables. For instance, although it was mentioned that ischemic heart failure patients spent more time engaging in vigorous activities, there was no clarification regarding the comparison group or the specific period during which this observation was made. Similarly, it was noted that sleep behaviour and physical activity were linked to patient-reported physical function, quality of life, and cognitive function. However, details such as the number of studies and participants for each specific association, the observation period, and the type of sleep behaviour observed remained unclear.

## Commentary

Overall, this systematic review is likely to provide an accurate and comprehensive summary of the results of the available studies addressing the question of interest. However, further research is needed to provide a more detailed and holistic review of this subject.

This review highlights the promising potential of using implantable accelerometer-based methods to identify the potential risks in patients with an ICD. Based on the current evidence in the review, there is insufficient evidence to support the use of accelerometers as a stand-alone detection method for increased risks of negative clinical outcomes (e.g. mortality & heart failure). However, they may serve as a moderating factor in a multifactorial assessment in identifying clinical deterioration.

Determining the precise impact of low or declining physical activity within a predictive model is challenging, as no single estimate of association was generated for any outcome in this systematic review. Therefore, to create an accurate weighted model, a weighted assessment of association (meta-analysis) for both low and declining physical activity across the outcomes assessed in this review is required. Individual estimates could then be integrated into multifactorial predictive models, alongside other key prognostic factors identified in previous systematic reviews. Previous studies in this area have identified additional factors associated with increased mortality risk, including anxiety and depression (Lindekilde et al. 2022), dialysis, chronic renal disease, cancer, advanced age (Alhakak et al. 2022), and ICD shocks (Qian et al. 2016). Accelerometer data on physical activity, if available, could enhance clinical risk stratification by serving as an additional indicator of potential clinical deterioration. This data can be combined with other key risk factors, such as anxiety, depression, and comorbidities, to provide a more comprehensive assessment of a patient's overall risk profile. Due to the significant inconsistencies across studies and insufficient reporting within the review, it is challenging to draw any clear recommendations or conclusions regarding the findings on wearable accelerometers.

Primary research in this area should aim to standardize both the exposed and unexposed groups, as well as establish consistent outcomes measured at multiple time points. Given the perceived but untested heterogeneity identified in this review, further investigation into potential moderating factors is recommended. Future systematic reviews should aim to focus on a single type of study—either aetiology or test accuracy. While combining these two assessments in one review is possible, it requires two distinct methodologies for critical appraisal and evidence synthesis. Separating them may also enhance search strategies by allowing for more specific term sets tailored to the review topic.

Additionally, future systematic reviews should aim for a more comprehensive search strategy, as the focus on only two databases in this review may have led to the omission of relevant studies. To improve data synthesis, future studies should attempt a meta-analysis when there is sufficient data commonality. If the data proves too heterogeneous for meta-analysis, alternative methods such as vote counting based on the direction of association, reporting medians and quartile ranges, or using visualization techniques like Harvest Plots, Albatross Plots, or Strip Charts should be employed. Given the methodological limitations of these approaches, findings should be interpreted with caution unless more robust synthesis methods are feasible. Moreover, future research must emphasize the complete and consistent reporting of all key variables to allow for a more accurate interpretation of the results.

CPD reflective questions

1. What are the key limitations of the systematic review presented in this commentary?
2. What advice can be given on lifestyle modifications and adherence to cardiac rehabilitation post-surgery to improve prognosis?
3. What are the other moderating factors of sudden cardiac death that should be considered in future research?

## Figures and Tables

**Table 1:** Critical appraisal using the JBI critical appraisal checklist for systematic reviews and research syntheses (delete the tool which is not relevant)

JBI critical appraisal checklist items	Responses
1. Which is not specifically a test accuracy	No - The review addresses a broad clinical question that does not specifically focus on diagnostic test accuracy or prognosis studies. With the review stating that the question of interest is “What is the clinical value of PB for identification of clinical deterioration?” rather than directly focusing it
2. Were the inclusion criteria appropriate for the review question?	Yes - The inclusion criteria are appropriate. Patients with an implantable cardioverter-defibrillator, regardless of the use of cardiac resynchronisation therapy or wearable cardioverter-defibrillator, were all included. Patients who are at high risk of developing sudden cardiac death without ICD were included. Eligible accelerometer-based methods included wearable or device-embedded accelerometery, with outcomes of interest being ICD therapy for ventricular arrhythmias, heart failure hospitalization, mortality, functional status (e.g., NYHA class), and quality of life. The included population allows the review to have a detailed comparison between ICDs and wearable accelerometer.
3. Was the search strategy appropriate?	Yes, the review used 2 electronic databases MEDLINE and EMBASE to identify studies published between Jan 2000 and Aug 2020. Relevant keywords and terms were used to search for papers, and reference lists of relevant papers were reviewed to identify potentially missed papers.
4. Were the sources and resources used to search for studies adequate?	No, this review only covers 2 electronic databases. This review should ideally cover multiple electronic databases, as well as trial registries to minimise the risk of publication bias.
5. Were the criteria for appraising studies appropriate?	No- QUIPS (Quality in Prognosis Studies) tool was used in the review to appraise the included studies. However, as indicated in the results, three studies explored validation of wearable and device-embedded accelerometery, and these validation studies should be assessed accordingly.
6. Was critical appraisal conducted by two or more reviewers independently?	Yes, the review stated that 2 independent reviewers conducted the appraisal. If there were any disagreement, a third reviewer will be consulted.
7. Were there methods to minimize errors in data extraction?	No - Data extraction was completed by two reviewers; however, it was not specifically stated whether the screening process was undertaken independently.
8. Were the methods used to combine studies appropriate?	No – The methods section lacks clear details on how the descriptive analysis was performed. It appears that a vote counting method was used. However, study direction was not presented as a ratio, making it difficult to assess the degree of association for all outcomes. Similarly, the results show consistency in the hazard ratios presented across the studies, although no explanation was provided for why a meta-analysis was performed.
9. Was the likelihood of publication bias assessed?	No, there was no specific reference used to assess publication bias.

## Key Points

- Declining physical activity in individuals with implantable cardioverter-defibrillators may be associated with an increased risk of mortality and hospitalisation.
- Individuals with implantable cardioverter-defibrillators and low physical activity may be at increased risk of mortality and hospitalisation.
- Future primary research should focus on standardizing the timing of outcome reporting and outcomes being assessed
